# Intermittent Hypoxia-Induced Carotid Body Chemosensory Potentiation and Hypertension Are Critically Dependent on Peroxynitrite Formation

**DOI:** 10.1155/2016/9802136

**Published:** 2015-12-20

**Authors:** Esteban A. Moya, Paulina Arias, Carlos Varela, María P. Oyarce, Rodrigo Del Rio, Rodrigo Iturriaga

**Affiliations:** ^1^Laboratorio de Neurobiología, Departamento de Fisiología, Facultad de Ciencias Biológicas, Pontificia Universidad Católica de Chile, 8330025 Santiago, Chile; ^2^Centro de Investigación Biomédica, Universidad Autónoma de Chile, 8900000 Santiago, Chile; ^3^Dirección de Investigación, Universidad Científica del Sur, Lima, Peru

## Abstract

Oxidative stress is involved in the development of carotid body (CB) chemosensory potentiation and systemic hypertension induced by chronic intermittent hypoxia (CIH), the main feature of obstructive sleep apnea. We tested whether peroxynitrite (ONOO^−^), a highly reactive nitrogen species, is involved in the enhanced CB oxygen chemosensitivity and the hypertension during CIH. Accordingly, we studied effects of Ebselen, an ONOO^−^ scavenger, on 3-nitrotyrosine immunoreactivity (3-NT-ir) in the CB, the CB chemosensory discharge, and arterial blood pressure (BP) in rats exposed to CIH. Male Sprague-Dawley rats were exposed to CIH (5% O_2_, 12 times/h, 8 h/day) for 7 days. Ebselen (10 mg/kg/day) was administrated using osmotic minipumps and BP measured with radiotelemetry. Compared to the sham animals, CIH-treated rats showed increased 3-NT-ir within the CB, enhanced CB chemosensory responses to hypoxia, increased BP response to acute hypoxia, and hypertension. Rats treated with Ebselen and exposed to CIH displayed a significant reduction in 3-NT-ir levels (60.8 ± 14.9 versus 22.9 ± 4.2 a.u.), reduced CB chemosensory response to 5% O_2_ (266.5 ± 13.4 versus 168.6 ± 16.8 Hz), and decreased mean BP (116.9 ± 13.2 versus 82.1 ± 5.1 mmHg). Our results suggest that CIH-induced CB chemosensory potentiation and hypertension are critically dependent on ONOO^−^ formation.

## 1. Introduction

The obstructive sleep apnea (OSA) syndrome, a worldwide sleep-breathing disorder, is recognized as an independent risk factor for hypertension [1–3]. OSA is characterized by repeated episodes of complete or partial obstruction of the upper airway during sleep, resulting in chronic intermittent hypoxic and hypercapnic events. Among the disturbances produced by OSA, chronic intermittent hypoxia (CIH) is considered the main factor for the development of systemic hypertension [1–4]. Although the link between OSA and hypertension is well known, the mechanisms responsible for the hypertension are not entirely understood. OSA elicits oxidative stress, inflammation, and sympathoexcitation, which contribute to the endothelial dysfunction and hypertension [1, 2, 5–8]. Recently, it has been proposed that the carotid body (CB), the main O_2_ chemoreceptor, plays a pivotal role in the development of the enhanced sympathetic activity and the generation of the hypertension following CIH [1, 2, 7, 8]. Indeed, CIH selectively enhanced CB chemosensory discharges during normoxia and hypoxia [8–12], which in turn led to a sustained potentiation of the sympathetic discharges to blood vessels, eliciting neurogenic hypertension [1, 2, 7, 12–14]. The repetitive episodes of hypoxia-reoxygenation during CIH produce oxidative stress due to the accumulation of reactive oxygen species (ROS) [1–7]. The evidence indicates that the CB chemosensory potentiation induced by CIH is mediated by oxidative stress, which increases the levels of CB excitatory modulators such as angiotensin II and endothelin-1, and reduces the bioavailability of the inhibitory chemosensory modulator nitric oxide (NO) in the CB [15, 16]. Peng et al. [8] proposed that the superoxide radical (O_2_^−^) participates in the potentiation of the rat CB chemosensory responses to hypoxia since they found that pretreatment of rats for 10 days before and concomitant with the exposure to CIH with manganese (III) tetrakis (1-methyl-4-pyridyl) porphyrin pentachloride (MnTMPyP), a superoxide dismutase (SOD) mimetic, prevented the CB chemosensory potentiation. We tested the hypothesis that oxidative stress contributes to the CB chemosensory potentiation and the progression of the hypertension in rats exposed to CIH [9]. We found that ascorbic acid treatment prevented systemic and local CB oxidative stress, the potentiation of CB chemosensory responses to hypoxia, and the hypertension in rats exposed to CIH for 21 days [9]. Although these results suggest that CB chemosensory potentiation is mediated by oxidative stress, it is matter of debate whether superoxide* per se* may increase the CB chemosensory discharges [17]. Thus, it is plausible that molecules downstream ROS formation may mediate the effects of oxidative stress on CB chemoreception. It is well known that O_2_^−^ radical reacts with NO to produce peroxynitrite (ONOO^−^), which can nitrate several proteins residues. Indeed, we have previously shown that CIH increases 3-nitrotyrosine immunoreactivity (3-NT-ir) levels in the CB and that changes in 3-NT-ir correlate with the enhanced CB chemosensory responses to hypoxia following CIH [18]. Accordingly, it is plausible that nitrooxidative stress, through an ONOO^−^ dependent pathway, may play a role in OSA pathophysiology. Thus, we studied if ONOO^−^ is involved in the enhanced CB chemosensitivity and the generation and maintenance of the hypertension induced by CIH. Therefore, we tested the effects of Ebselen treatment, a potent ONOO^−^ scavenger [19–21], on 3-NT-ir accumulation in the CBs, CB chemosensory responses to hypoxia, and arterial blood pressure (BP) in conscious rats exposed to CIH, a well stablished experimental model of OSA [7, 9, 15, 18].

## 2. Material and Methods

### 2.1. Animals and Intermittent Hypoxia Protocol

Experiments were performed on adult male Sprague-Dawley rats weighting 200 g fed with standard diet ad libitum and kept on a 12:12-hour light dark cycle. Room temperature was maintained between 23 and 25°C. All the experimental procedures were approved by the Bioethical Committee of the Biological Sciences Faculty, Pontificia Universidad Católica de Chile, Santiago, Chile, and were performed according to the National Institutes of Health Guide (NIH, USA) for the care and use of animals. Unrestrained, freely moving rats were housed in individual chambers (12 cm × 35 cm, 3 L) and exposed to hypoxic cycles of 5% inspired O_2_ for 20 s, followed by 280 s of room air, 12 times per hour during 8 hours a day, or exposed to sham air-air cycles, emulating the same conditions of noise, temperature, and flow [7, 9, 10]. Rats were exposed to CIH from 8:00 AM to 16:00 PM. The O_2_ level inside the chambers was continuously monitored with an oxygen analyzer (Ohmeda 5120, BOC Healthcare, Manchester, UK) and the CO_2_ levels and humidity were maintained at low levels by continuous air extraction.

### 2.2. Carotid Body Chemosensory Recording

The CB chemosensory discharges were measured* in situ* as previously described [9, 10, 12]. Rats were anesthetized with sodium pentobarbitone (40 mg/kg) and additional doses were given to the animal when necessary to maintain a level of surgical anesthesia. Rats were placed on supine position and the body temperature was maintained at 38 ± 0.5°C with a heating pad. The trachea was cannulated for gases administration. One carotid sinus nerve was dissected and placed on a pair of platinum electrodes and covered with mineral oil. The neural signal was preamplified (Grass P511, Grass Instruments, Quincy, MA, USA), filtered (30 Hz–1 kHz), and fed to an electronic spike-amplitude discriminator, allowing the selection of action potentials of particular amplitude above the noise to be counted with a frequency meter, thus being able to measure the frequency of carotid chemosensory discharges (*f*_*x*_) expressed in Hz. The carotid sinus electroneurogram was continuously monitored using an oscilloscope. Baroreceptor activity, which consisted of repetitive and rhythmic discharges synchronized with the systolic arterial blood pressure, was eliminated by crushing the baroreceptor fibers between the carotid bifurcation and the CB. This procedure resulted in the complete elimination of discharge synchronized with arterial blood pressure. 100% O_2_ (Dejours test) was used to confirm that all the neural activities recorded correspond to chemosensory discharge [9, 12]. The contralateral carotid sinus nerve was cut to prevent vascular and ventilatory reflexes evoked by hypoxic activation. The CB chemosensory frequency of discharge (*f*_*x*_) was measured in response to several levels of inspired PO_2_ (~5–670 mmHg, applied for 20–30 s), by averaging the maximal values during the semiplateau of the chemosensory response. Thus, *f*_*x*_ is the absolute value of chemosensory rate of discharge. The O_2_ levels were measured with an oxygen analyzer (Ohmeda 5120, BOC Healthcare, Manchester, UK).

### 2.3. Arterial Blood Pressure Telemetry

In a subset of rats, arterial blood pressure (BP) was recorded using radio-telemetry. Briefly, rats were anesthetized with 5% isoflurane and maintained with 1-2% isoflurane in 100% O_2_ during the surgical procedure. An abdominal incision was performed to isolate the abdominal aorta. The tip of a cannula-coupled telemetry device (Telemetry Research TRM54P, Millar USA) was inserted into the abdominal aorta and fixed with methacrylate. After this procedure, the abdominal incision was sutured in layers. At the end of the surgery, rats received an i.p. injection of enrofloxacin (1%) and ketoprofen (1%) and were supplied during 3 subsequent days with the same dose of enrofloxacin in the drinking tap water. Physiological variables were acquired with an analogue-digital system (PowerLAB 8SP, ADInstruments, Australia) and analyzed with the Chart 7.2-Pro software. BP was measured after one week of recovery. For quantification of the baseline BP during normoxia, we averaged 10 minutes of BP signal, recorded 30 minutes before the beginning of the CIH protocol. To quantify ΔBP response evoked by acute 5% O_2_, we measured the difference between the maximal BP averaged for 5 s and the baseline BP values averaged for 1 min before the acute hypoxic challenge.

### 2.4. Experimental Procedure and Ebselen Administration

The peroxynitrite targeted antioxidant Ebselen (Enzo Life Sci, Inc., Farmingdale, NY, USA) was administrated with osmotic minipumps (2ML4, Alzet Scientific Products, Chevy Chase, MD, USA). Rats were anesthetized with isoflurane in 100% O_2_, and osmotic minipumps were implanted subcutaneously on the back. Pumps were filled with 33.3 mg Ebselen in 1 mL of 80% DMSO in saline, to achieve a delivering rate of 10 mg/kg/day, a similar dose used in other studies [22, 23]. Control animals were implanted with osmotic minipumps filled with DMSO 80% in saline solution. After surgical procedures, the rats were treated with enrofloxacin and ketoprofen as mentioned before. Due to the nature of the experiments related to the study of CB chemosensory activity, a cross-sectional study was performed. Twenty-four rats were randomly divided into 3 groups: one control group, exposed to sham conditions and treated with Ebselen (Ebselen-Sham); a second group, exposed to CIH and treated with vehicle (Vehicle-CIH); and a third group, exposed to CIH and treated with Ebselen during the hypoxic protocol (Ebselen CIH). To study the therapeutic effect of Ebselen on the CIH-induced hypertension, we performed a longitudinal study. Rats with indwelling catheter from telemetry devices were exposed first to sham conditions for 7 days and then to CIH for other 7 days. At the end of the CIH 7-day exposure, an osmotic minipump filled with Ebselen was subcutaneously implanted and the rats were kept another week in CIH.

### 2.5. Nitrotyrosine Immunohistochemistry

At the end of the CB chemosensory studies, anesthetized rats were perfused intracardially with saline at pH 7.4 for 15 min followed by buffered 4% paraformaldehyde (PFA, Sigma, St. Louis, MO, USA). The carotid bifurcations were dissected and postfixed in the same fixative solution for 12 h to 4°C. Then, the samples were dehydrated in ethanol, included in paraffin, cut in 5 *μ*m sections, and mounted on silanized slides. Samples were deparaffinized and exposed to antigen-retrieval solution (citrate buffer 1 M, pH 6.0) as previously described [9, 18]. Samples were then incubated in 0.3% H_2_O_2_ solution and blocked using normal horse serum (ABC, Vectastain kit, Vector) for 1 hour. The samples were then incubated with primary antibody against 3-NT (1 : 500, number A21285, Molecular Probes) overnight at 4°C. The immunoreactivity staining was detected using a streptavidin-peroxidase kit (ABC, Vectastain kit, Vector) and revealed at 37°C in dark chamber with 3,3′-diaminobenzidine tetrahydrochloride (DAB, Sigma). Samples were counterstained with Harris haematoxylin and mounted with Entellan (Merck, Whitehouse station, NJ, USA). Photomicrographs were taken at 100x using a CCD camera coupled to an Olympus CX 31 microscope (Olympus Corp., USA), digitized, and analyzed using ImageJ software (NIH, Betheseda, MD, USA). We measure two nonconsecutive CB sections per rat, obtaining four CB photographs from each one of those sections. The positive 3-NT-ir, averaged from the eight CB fields, was expressed as optical integrated intensity, in arbitrary units.

### 2.6. Statistical Data Analysis

**Figure 1:**
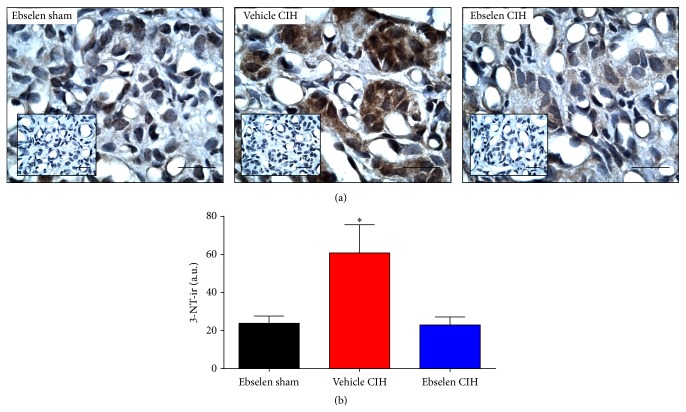
Ebselen treatment prevented the increased levels of 3-NT-ir. (a) Representative effects of Ebselen on positive 3-NT immunoreactivity (3-NT-ir) in CBs from rats exposed to CIH. Inset, negative controls omitted inclusion of primary antibody. Scale bars 20 *μ*m. (b) Summary of the effects of Ebselen on 3-NT-ir measured in CBs. ^*∗*^ *P* < 0.05, vehicle CIH versus Ebselen sham and Ebselen CIH, and Bonferroni after one-way ANOVA, *n* = 5 rats per group.

**Figure 2:**
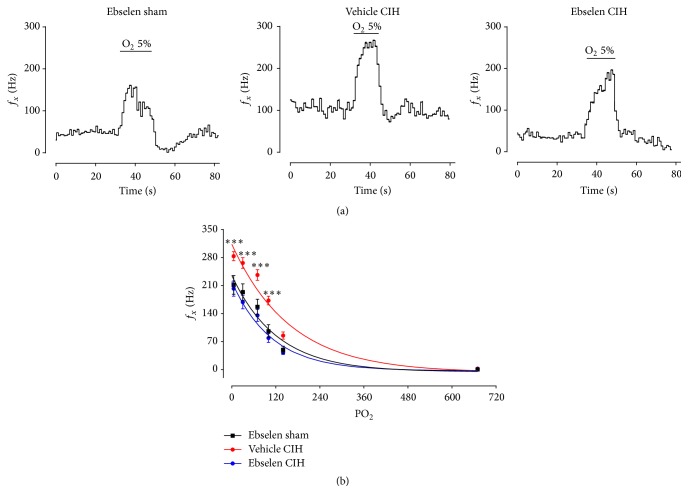
Ebselen treatment prevented the CB chemosensory potentiation in rats exposed to CIH. (a) Recordings of the CB frequency of chemosensory discharge (*f*_*x*_) measured from the carotid sinus nerve in response to acute hypoxia (5% O_2_) in rats treated with Ebselen or vehicle and exposed to sham or CIH conditions. The enhanced CB chemosensory response induced by CIH (vehicle CIH) was prevented by Ebselen (Ebselen CIH). (b) Summary of the effect of the Ebselen treatment at different levels of inspired PO_2_ (mmHg) on *f*_*x*_. ^*∗∗∗*^ *P* < 0.001, vehicle CIH versus Ebselen sham and Ebselen CIH, and Bonferroni after two-way ANOVA, *n* = 8 rats per group.

**Figure 3:**
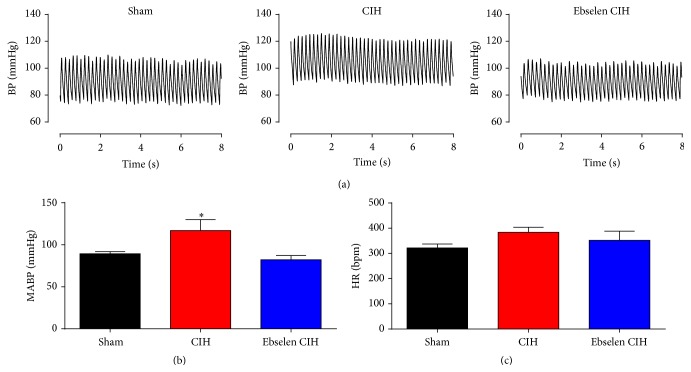
Ebselen reversed the increased arterial blood pressure measured during normoxia in CIH rats. Telemetric recording realized in the same rat during control condition ((a), sham), after 7 days of CIH ((a), CIH) and the effect of Ebselen after additional 7 days of CIH ((a), Ebselen CIH). Summary of the effect of Ebselen on mean arterial blood pressure (MABP, (b)) and heart rate (HR, (c)). ^*∗*^ *P* < 0.05, CIH versus sham and Ebselen CIH, and Newman-Keuls after Repeated Measures ANOVA, *n* = 4 rats.

**Figure 4:**
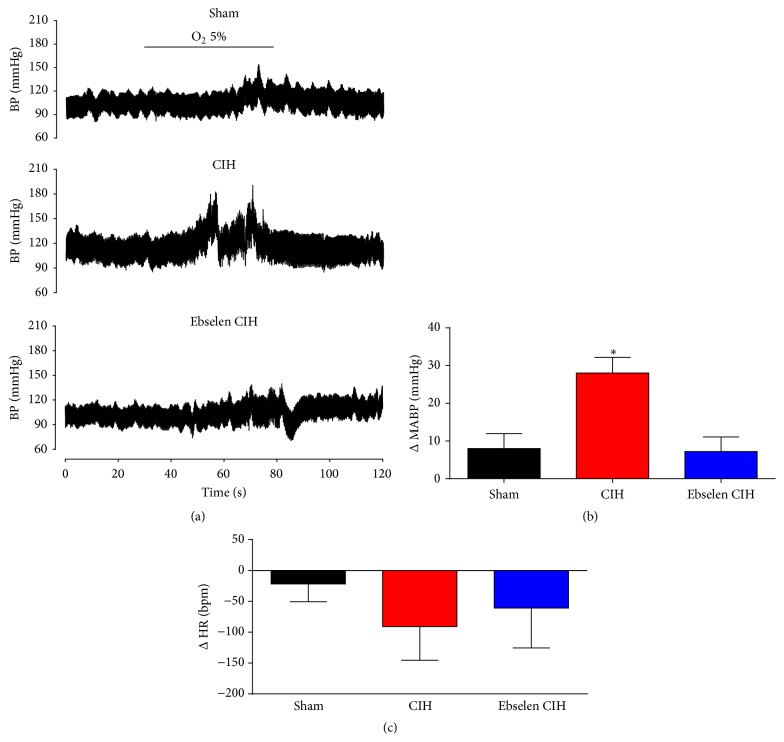
Ebselen reversed the CIH-induced increase in BP responses evoked by an acute hypoxic stimulus. Telemetry recordings realized in the same rat show the pressure response evoked by hypoxia (5% O_2_ for 50 s), during sham conditions, after 7 days of CIH, and after additional 7 days of CIH but supplemented with Ebselen (a). Summary of the effect of Ebselen on mean arterial blood pressure (MABP, (b)) and heart rate (HR, (c)). ^*∗*^ *P* < 0.05, CIH versus sham and Ebselen CIH, and Newman-Keuls after Repeated Measures ANOVA, *n* = 4 rats.

Data was expressed as mean ± SEM. For cross-sectional studies (Figures 1 and 2), statistical analysis was performed using one-way or two-way ANOVA tests followed by Bonferroni* post hoc* analysis. For longitudinal studies (Figures 3 and 4 and Tables 1 and 2), Repeated Measures one-way ANOVA followed by Newman-Keuls* post hoc* comparisons was used. *P* < 0.05 was set as the level of statistical significance for both studies.

## 3. Results

### 3.1. Effects of Ebselen on the CIH-Induced Increase of 3-NT-ir in the CB

The exposure to CIH for 7 days produced a marked increase in the 3-NT-ir levels in the CB (Figure 1(a)). Indeed, we found a 2.5-fold increase in 3-NT-ir in the CB from CIH-treated rats compared to the levels observed in sham rats. The administration of the peroxynitrite scavenger Ebselen to the rats during the CIH exposure prevented the CIH-induced increase of 3-NT-ir in the CB (Figure 1(b)). Figure 1(b) shows the quantification of the effects of Ebselen on the 3-NT-ir accumulation induced by CIH. Rats exposed to CIH and treated with Ebselen showed 60% of reduction in 3-NT-ir as compared with the CIH rats treated with vehicle (60.8 ± 14.9 versus 22.9 ± 4.2 a.u., *P* < 0.05, and CIH and CIH Ebselen rats, resp.).

### 3.2. Ebselen Prevented CB Chemosensory Potentiation Induced by CIH

To assess the effect of Ebselen on CB chemosensory activity, we measured the frequency of chemosensory discharge (*f*_*x*_), from the carotid sinus nerve from rats exposed to CIH and treated with Ebselen (Figure 2). The exposure to CIH for 7 days increases the baseline CB chemosensory and the discharge evoked by hypoxia. Indeed, Ebselen treatment prevented the potentiation of the hypoxic CB chemosensory response in CIH rats (Figure 2). The two-way ANOVA analysis showed a significant increase of CB chemosensory discharge for different levels of inspired PO_2_ in rats exposed to CIH (*P* < 0.01). The treatment with Ebselen during CIH exposure effectively prevented the CB increased responses to several levels of hypoxia (Figure 2(b)).

### 3.3. Effects of Ebselen on the CIH-Induced Hypertension

Exposure to 7 days of CIH produced a significant increase in baseline BP measured in normoxia (Figure 3). We found that, after one week of CIH exposure, the mean arterial blood pressure (MABP) increased about 25 mmHg compared to the value measured during sham condition (89.3 ± 2.5 mmHg versus 116.9 ± 13.2 mmHg, *P* < 0.05 sham versus CIH, resp.). Remarkably, Ebselen treatment normalized MABP during CIH to similar levels to those observed during sham conditions (82.1 ± 5.1 mmHg, Figure 3). The values for baseline systolic (*P*_*s*_), diastolic (*P*_*d*_), and pulse pressure (*P*_*p*_) are summarized in Table 1. We did not find significant differences in resting heart rate (HR) between animals exposed to sham, CIH, and Ebselen CIH conditions (sham 321.9 ± 15.5, CIH 383.6 ± 20.3, and CIH Ebselen 351.8 ± 36.3 beats per minute, Figure 3(c), *P* > 0.05, one-way ANOVA). In a separate experimental series, we measured MABP and HR in 3 sham rats after one week of the implantation of osmotic pumps containing the vehicle (DMSO 80%). We did not find any differences (MABP 97.4 ± 3.9 mmHg, HR 333.1 ± 5.3 beats per minute) related to the values recorded in rats implanted with pumps containing Ebselen in DMSO in sham conditions for one week.

In addition, we measured the BP response evoked by acute hypoxia (Figure 4). Acute hypoxic episodes (5% O_2_) in sham rats produced a mild increase in BP (ΔMABP = 8.0 ± 3.9 mmHg). In contrast, after one week of CIH exposure, the BP response to the same level of hypoxia was largely increased (ΔMABP = 28.1 ± 4.1 mmHg). Ebselen treatment during CIH exposure normalized BP responses to hypoxia (ΔMABP = 7.2. ± 3.9 mmHg). The mean values for Δ*P*_*s*_, Δ*P*_*d*_, and Δ*P*_*p*_ during acute hypoxic stimulus are shown in Table 2. Therefore, treatment with Ebselen effectively restores the normal arterial pressure response to hypoxia, even in the presence of CIH. The increases in BP following acute hypoxic stimulation produced a reflex bradycardia in all three conditions (sham, CIH, and Ebselen CIH). The ΔHR response to hypoxia did not reach statistical significance between the treatments (*P* > 0.05, Sham = 21.8 ± 28.8, CIH = 90.8 ± 54.5, and Ebselen CIH = 60.8 ± 64.8 beats per minute, Figure 4(c), *P* < 0.05, one-way ANOVA).

## 4. Discussion

Present results show that Ebselen prevented the accumulation of 3-NT-ir in the CB and the enhanced CB chemosensory discharges induced by CIH. Indeed, Ebselen reduced the baseline chemosensory discharge and the responses to hypoxia (Figure 2), confirming observations showing that antioxidant treatment prevents the potentiation of the rat CB chemosensory response to hypoxia induced by CIH [8, 9]. Furthermore, we found that administration of Ebselen, once rats already developed hypertension induced by CIH exposure, was able to normalize baseline BP in normoxia and the BP response to acute hypoxia. Our results suggest that increases in ONOO^−^ in the CB contribute to the CB chemosensory potentiation induced by CIH. In addition, CIH-induced systemic hypertension is critically dependent on ONOO^−^ since Ebselen treatment reduces BP to values similar to the ones measured in normotensive animals. Thus, it is plausible that the primary action of Ebselen reduced the exacerbation of CB chemosensory output and the sympathetic induced hypertension, but we cannot exclude other effects on the hypoxic chemoreflex pathway. To our knowledge, this is the first study that shows that an ONOO^−^ scavenger was effective to prevent the CB chemosensory potentiation and reverses the hypertension induced by CIH.

A growing body of evidence supports the proposal that the CB contributes to the autonomic dysfunction and hypertension in OSA patients and animals exposed to CIH. Indeed, patients with recently diagnosed OSA show enhanced ventilatory, pressor, and sympathetic responses to acute hypoxia, attributed to a potentiation of the CB chemoreflexes [1, 2, 7]. Indeed, Narkiewicz et al. [13] found potentiated reflex ventilatory, tachycardic, and pressor responses to acute hypoxia in untreated normotensive patients with OSA. On the contrary, the ventilatory and pressor responses induced by hypercapnia and by the cold pressor test in OSA patients were not different from those observed in control subjects. Similarly, animals exposed to CIH show enhanced hypoxic ventilatory responses to acute hypoxia [7] for review and long-term facilitation of respiratory motor responses [8, 11]. Recording of chemosensory discharges from the carotid sinus nerve has confirmed the idea that CIH produces facilitation of the CB chemosensory responses to hypoxia. Indeed, exposure of rats and cats to CIH for few days increases the baseline CB discharges measured in normoxia and enhances the chemosensory responses to acute hypoxia [8–12]. Peng et al. [8] reported that baseline CB discharge and chemosensory responses to acute hypoxia were higher in rats exposed to short cyclic hypoxic episodes followed by normoxia, applied during 8 hours for 10 days. Similarly, we found that cats and rats exposed to CIH for 7 days showed enhanced CB chemosensory and ventilatory responses to acute hypoxia [8, 12]. Studies performed in OSA patients and animals exposed to CIH show that OSA is associated with sympathoexcitation, mainly attributed to the enhance CB chemosensory function elicited by CIH [1, 2, 7].

Several studies have proposed that ROS are involved in the progression of the cardiovascular pathologies in patients suffering OSA and animals exposed to CIH [2, 3, 5, 7, 24]. Indeed the O_2_^−^ radical has been proposed as the main ROS responsible for these pathological consequences, since treatment with SOD mimetic prevented the hypertension induced by CIH in rats [8, 24]. It is well known that O_2_^−^ reacts with nitric oxide (NO) producing ONOO^−^ with an elevated constant rate of ~7·10^9^/M s [25]. Interestingly, this rate is 3.5 times higher than its enzymatic dismutation by SOD [26]. This fast reaction explains how these particularly elusive species could rapidly react to form ONOO^−^ [27], reducing the NO bioavailability [28, 29]. Accordingly, we previously found a reduction in the NO production in the rat CB after 7 days of CIH [30]. Since NO is considered an inhibitory modulator of CB chemosensory discharges [31], a reduced NO level may partially contribute to enhancing the baseline CB discharges and chemosensory responses to hypoxia. This interpretation agrees with the observation of Marcus et al. [32], who found that CIH decreased the expression of the nNOS in the rat CB, suggesting that the removal of the normal inhibitory NO influence contributes to enhancing the CB chemosensory responses to hypoxia.

There is evidence suggesting that ONOO^−^ radical is involved in the development of diseases such as type I diabetes, cancer, stroke, heart failure, and neurodegenerative disorders [33, 34]. The ONOO^−^ radical is highly unstable and produces deleterious reactions and cytotoxic effects, such as oxidation of several molecular targets like lipids, proteins, and DNA [35–37]. One of the main consequences of increased levels of ONOO^−^ is the modification of tyrosine residues in proteins producing 3-NT [27], which has been related with many diseases and cellular damage including liver disease [38], chronic allograft nephropathy [39], and Alzheimer's and Parkinson's disease [40]. We found an increase of 3-NT-ir accumulation in the CB from rats exposed for 7 to 21 days to CIH, suggesting that ONOO^−^ formation due to the reaction of NO with O_2_^−^ is a critical step in the CB chemosensory potentiation induced by CIH [9, 18]. Present results agree with and extend the idea that ONOO^−^ radical contributes to the CB enhanced responses to hypoxia after CIH. Ebselen is an organoselenium compound that mimics glutathione peroxidase activity [19–21], which rapidly reacts with ONOO^−^ [19]. The generation of 3-NT is a direct result of the ONOO^−^ generation, and the treatment with Ebselen prevented the CIH-induced increase of 3-NT-ir levels in the CB and the chemosensory potentiation of the CB after CIH, suggesting that protein nitration may play a role in enhancing the chemoreceptor responses to acute hypoxia.

To study plausible therapeutic effects of Ebselen in an experimental model of OSA, we decide to administrate Ebselen after the development of hypertension in rats exposed to CIH. We found that Ebselen treatment effectively normalized resting BP in awake rats, even in the presence of the intermittent hypoxic stimulus (Figure 3). In addition, Ebselen abolished the potentiated BP response to acute hypoxic stimulation observed during CIH exposure (Figure 4). Taken together, Ebselen administration should be considered as a novel tool to restore normal BP adjustments following CIH. In contrast, we did not find any significant difference between the HR in response to acute hypoxia between the treatments (Figure 4). Acute hypoxia in conscious rats generates a biphasic HR response, characterized by an initial tachycardia during mild hypoxia, but bradycardia when inspired fraction of O_2_ decrease below 8% [41]. Then, chronic exposure to intermittent hypoxia may affect both the tachycardic and bradycardic responses to acute hypoxia. Future studies are needed to elucidate these questions.

## 5. Limitations of the Study

Our results suggest that Ebselen prevents the nitration of proteins in the CB, which contributes to normalizing the CB frequency of discharge and the sympathetic-mediated hypertension. Administration of Ebselen was achieved using subcutaneous osmotic minipumps; therefore, the treatment is delivered systemically. Thus, it is possible that Ebselen may act not only in the CB, but also in other parts of the chemosensory pathway (i.e., nucleus of the tractus solitary, rostral ventrolateral medulla), since Ebselen can cross the blood brain barrier [42]. Rats exposed to CIH show increased plasma renin activity [43], and it is known that Losartan treatment prevents the CIH-induced hypertension [43], the increased sympathetic activity induced by apnea episodes [32], and the decrease in arterial vasodilation induced by acetylcholine after 28 days of CIH [44]. Moreover, intracerebroventricular injection of Losartan prevents the hypertension induced by CIH and the neuronal activation in areas related to sympathetic activation [45]. Thus, we cannot exclude effects of Ebselen at the central nervous system, sympathetic peripheral system, or arterial vessels, which may all be involved in the antihypertensive effect of Ebselen.

## 6. Conclusion 

Present results suggest that 3-NT accumulation contributes to the CB chemosensory potentiation through the nitration of protein residues, which in turn promotes hypertension. Ebselen treatment prevents the increased CB chemosensory activity and reverses the hypertension in rats exposed to CIH, suggesting that the CB chemosensory potentiation plays a key role in the generation and maintenance of the hypertension induced by CIH. Further development of ONOO^−^ targeted scavengers should be of therapeutic interest in the treatment of hypertension in OSA patients.

## Figures and Tables

**Table 1:** Systolic, diastolic, and pulse pressure measured in normoxia in the same rats exposed to sham, CIH, or Ebselen CIH condition.

	Sham	CIH	Ebselen CIH
*P*_*s*_ (mmHg)	109.7 ± 2.7	138.0 ± 12.6^*∗*^	110.0 ± 3.5
*P*_*d*_ (mmHg)	79.1 ± 2.7	106.4 ± 13.5^*∗*^	71.2 ± 4.9
*P*_*p*_ (mmHg)	30.6 ± 1.4	31.7 ± 2.0	32.9 ± 3.0

*P*_*s*_, systolic, *P*_*d*_, diastolic, and *P*_*p*_ pulse arterial pressure (*P*_*s*_ − *P*_*d*_). ^*∗*^ *p* < 0.05, CIH versus sham and Ebselen CIH and Newman-Keuls after Repeated Measures ANOVA, *n* = 4 rats.

**Table 2:** Arterial blood pressure responses to 5% O_2_ measured during normoxia in the same rats exposed to sham, CIH, or Ebselen CIH condition.

	Sham	CIH	Ebselen CIH
Δ*P*_*s*_ (mmHg)	14.6 ± 3.5	45.5 ± 4.6^*∗*^	14.4 ± 5.7
Δ*P*_*d*_ (mmHg)	4.7 ± 4.3	19.7 ± 4.3	3.6 ± 3.5
Δ*P*_*p*_ (mmHg)	9.9 ± 1.9	17.2 ± 10.3	10.8 ± 4.2

Δ*P*_*s*_, max-baseline systolic arterial pressure, Δ*P*_*d*_, max-baseline diastolic arterial pressure, and Δ*P*_*p*_, max-baseline pulse arterial pressure. ^*∗*^ *p* < 0.05, CIH versus sham and Ebselen CIH and Newman-Keuls after Repeated Measures ANOVA, *n* = 4 rats.
